# Supplementary material for: A UMLS-based spell checker for natural language processing in vaccine safety
Source: BMC Med Inform Decis Mak. 2007 Feb 12;7:3. doi: 10.1186/1472-6947-7-3 (PMC1805499; doi:10.1186/1472-6947-7-3)
Supplement: Additional file 13 — RAP application directory. Sets up directory for RAP (RDF application for PHP) source code [file 1472-6947-7-3-S13.gz › rap/doc/phpdoc/__filesource/fsource_default__rdqlRDQL.php.html]

xml version="1.0" encoding="iso-8859-1"?


File Source for RDQL.php


# Source for file RDQL.php

Documentation is available at RDQL.php

```
1. <?PHP
2. // ----------------------------------------------------------------------------------
3. // RDQL
4. // ----------------------------------------------------------------------------------
5. //
6. // Description               : Rdql package
7. // ----------------------------------------------------------------------------------


10. // include RDQL classes

12. require_once( RDFAPI_INCLUDE_DIR . 'rdql/RdqlParser.php' );
13. require_once( RDFAPI_INCLUDE_DIR . 'rdql/RdqlEngine.php' );
14. require_once( RDFAPI_INCLUDE_DIR . 'rdql/RdqlDbEngine.php' );
15. require_once( RDFAPI_INCLUDE_DIR . 'rdql/RdqlMemEngine.php' );
16. require_once( RDFAPI_INCLUDE_DIR . 'rdql/RdqlResultIterator.php' );

18. ?>
```

Documentation generated on Fri, 17 Dec 2004 16:17:24 +0100 by phpDocumentor 1.3.0RC3
